# Microchimerism in Graves' Disease

**DOI:** 10.1155/2012/724382

**Published:** 2012-04-05

**Authors:** Juan C. Galofré

**Affiliations:** Department of Endocrinology and Nutrition, University Clinic of Navarra, University of Navarra, Pio XII 36, 31080 Pamplona, Spain

## Abstract

Microchimerism is the presence of cells from one individual in another genetically distinct individual. Pregnancy is the main cause of natural microchimerism through transplacental bidirectional cell trafficking between mother and fetus. The consequences of pregnancy-related microchimerism are under active investigation. However, many authors have suggested a close relationship linking fetal microchimerism and the development of autoimmune diseases. It has been more than ten years now since the demonstration of the presence of a significant high number of fetal microchimeric cells residing in thyroid glands from operated patients with Graves' disease. This intrathyroidal fetal microchimerism is an attractive candidate mechanism for the modulation of Graves' disease in pregnancy and the postpartum period.

## 1. Introduction

 Microchimerism is defined by the presence of alien cells within an individual tissue with genetically different background [1]. Microchimeric cells have two possible origins: natural and artificial. Examples of the former are pregnancy, miscarriage, and twinning or sexual intercourse, whereas the most common cases for the later are tissue transplant or blood transfusion. Pregnancy is the major source of natural microchimerism.

 Contrary to previous expectations, the placental trophoblastic physical barrier effect is not a perfect cutoff system. A certain cell leakage is present between mother and fetus during gestation, and this transplacental cell trafficking is a two-way process. Fetal cells movement into maternal circulation starts very early during pregnancy. Circulating fetal cells have been found in maternal blood as soon as the fourth week of gestation [2], whereas maternal microchimerism has been detected in a newborn thyroid autopsied at day 2, but so far has not been reported in thyroid diseases [3]. Earlier evidences concluded that fetal cells transfer into maternal circulation was more intense than maternal cells into fetal blood [4]. However, subsequent investigations estimated that fetus-to-maternal transfer should be as frequent as maternal-to-fetus trafficking because maternal DNA has been detected in 40–100% of cord blood samples when polymerase chain reaction (PCR) techniques were used [5, 6]. The extent of this phenomenon is universal since fetal cells can be found in the peripheral blood of almost 100% of women during pregnancy [7]. Although the level of circulating cells has been reported to be very low (1 : 500,000 fetal : maternal cells) [8], such fetal cells can remain after delivery for more than 38 years postpartum [9]. The most plausible explanation for this long cell persistence is that fetal microchimeric cells can engraft into maternal bone marrow and provide a renewing source of fetal cells in maternal blood for decades after delivery [10].

**Figure 1:**
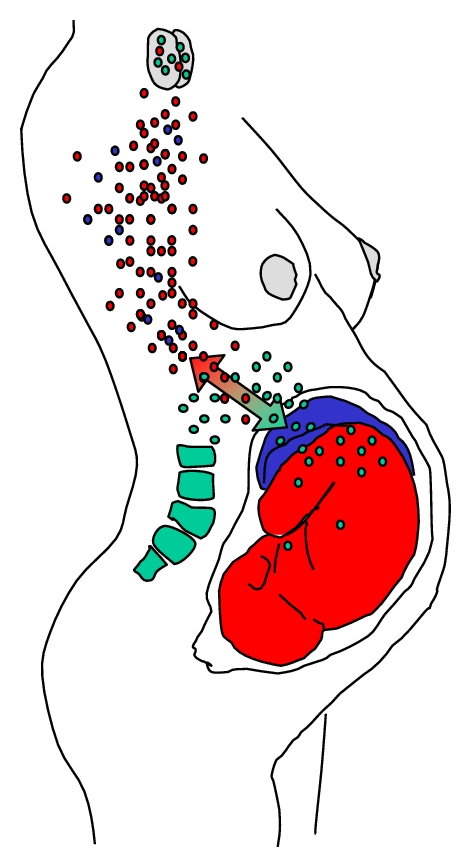
Pregnancy is the major source of natural microchimerism. Pregnancy related microchimerism results from deficiencies in the natural placental physical barrier tissue that divide the maternal circulation from the fetal circulation. As a consequence, there is mutual and bidirectional maternal and fetal cell traffic during pregnancy. Microchimeric cells enter the circulation and persist for many years in the host tissues. These cells are tolerated while acquire specific and diverse biological actions. (Copy of Figure 5 in [1]).

 So far, only the human leukocyte antigen (HLA) compatibility between mother and fetus has been identified as a factor influencing the persistence of microchimeric cells. Microchimerism and chronic graft versus host disease resemble each other. Both diseases share many clinical and pathological features with autoimmune diseases. Since autoimmune disease are more frequent in childbearing age females, it has been hypothesized that fetal microchimerism may be involved in their etiology. For a successful pregnancy, the maternal immune system must not overreact to the fetus. Dramatic changes throughout gestation make possible maternal tolerance of the fetus and permit fetal cells to move into maternal circulation and settle in maternal tissues. As a result, maternal tolerance allows the persistence of fetal microchimerism (Figure 1).

 Intrathyroidal fetal microchimerism has been reported using different techniques. PCR-based analysis identifies Y-chromosomal and merely demonstrates the presence of male cells in maternal tissues [11, 12]. Other methodologies, such as immunohistochemistry, fluorescence in situ hybridization (FISH) and HLA typing identify location, cellular progeny, and immunogenic properties of microchimerism in different tissues [13, 14].

## 2. Effects of Microchimerism

 According to cellular characterization, fetal microchimeric cells could cover a wide spectrum of action. Experimental data support a variety of important hypotheses concerning their biological implications. The types of cells crossing the placenta into the mother include both immune cells and cytokeratin-positive epithelial cells. Such cells have been identified as hematopoietic progenitor cells, nucleated erythrocytes, trophoblast cells, and leukocytes [15]. Therefore, microchimeric cells could potentially operate as effectors' cells or as targets of an immune response. Other possibilities include reactivity of microchimeric T-cell clones to the nonshared maternal human leukocyte antigen (HLA) antigens and presentation of microchimeric peptides by one host cell to another host cell [16].

 We have previously proposed a three-role division for fetal microchimerism, which covers *pathogenic*, *beneficial,* and *neutral* microchimerism [1]. The concept of *pathogenic microchimerism* initially suggested by Nelson [17], hypothesizes that fetal cells following gestation may lead to a graft versus host-like reaction in women. Accordingly, maternal immune response to these foreign cells may support an autoimmune reaction. It is also plausible the existence of a *beneficial microchimerism*, where persistent fetal cells may have a beneficial effect as a new source of progenitor cells potentially capable to contribute to maternal tissue repair processes. The third possibility could be *neutral microchimerism*, where fetal cells may act as innocent bystanders playing no role in biology at all.

 In line with our proposed microchimeric fetal cells varied effects, Fugazzola et al. [10] have recently speculated about three new possible roles of these foreign cells in relation with cancer, which are cell cancer destruction, tissue repair, and promotion of cell cancer progression. Apparently, a given fetal cell could act differently according to the particular tissue environment or depending on the type of malignancy.

## 3. Immune Changes during Pregnancy

 For a successful pregnancy, the maternal immune system must not overreact to the fetus. The mechanisms through which the immune systems between mother and fetus interact to induce and maintain this immune tolerance are not fully understood, but several changes have been described. Trophoblast cells serve both as physical barrier as well as immune modulator by expressing several molecules and secreting specific cytokines. This involves the expression of Fas-ligand, cytokines, indoleamine 2,3-dioxygenase, immune-modulating sex steroids, and HLA-G. Fas-ligand is involved in the removal of maternal T-cell clones that react to fetal antigens. In animal models, indoleamine 2,3-dioxygenase catabolizes tryptophan from maternal immune cells in the placental area, which is essential for a successful pregnancy in murine models, whereas HLA-G, exclusively expressed on trophoblast cells, inhibits natural killer cell-mediated cellular immunity [11].

 These changes also include a reduction of T-regulatory cells activity and affect maternal T-helper (Th) cell differentiation. Placental immune modulation promotes suppression of Th1 (cellular), whereas relative enhancement of Th2 (humoral) immunity occur. Thus placental immune suppression helps establish fetal microchimerism. Immune tolerance to fetal implant allows pregnant woman to accept fetal circulating cells [14]. Consequently, once fetal cell migrate and take up residence in maternal tissues, they may survive. This immune suppression may remain some months after delivery [18], allowing fetal cells to establish themselves and to survive the postpartum period [19]. Such dramatic changes throughout gestation make possible maternal tolerance of the fetus and permit fetal cells to move into maternal circulation and settle in maternal tissues. As a result, maternal tolerance allows the persistence of fetal microchimerism.

 This complex modulation of the maternal immune system by pregnancy is also reflected by the variation of circulating levels of autoantibodies. Furthermore, there is compelling evidences that circulating thyroid autoantibodies are predictors for the increased risk of pregnancy complications like miscarriage, breech presentation, or prematurity [20]. Probably thyroid autoimmunity represents a more profound abnormal immune state, which induces an unstable implant [21]. For instance, a recent Italian study showed that 37.2% of thyroid peroxidase (TPO) antibody-positive women had postpartum thyroiditis, versus 1.7% of the TPO antibody-negative women. Furthermore, 20% of the TPO antibody-positive women remained hypothyroid at the end of the first postpartum year versus 1% of TPO antibody-negative women [22]. Another classic study described that up to 60% of reproductive Graves' disease women reported the onset of the disease within oneyear after delivery [23].

 At the same time, it has been hypothesized that pregnancy-related immune changes have important positive effects for the fetal immune system. There is evidence suggesting that maternal immune cells instruct fetal cells how to balance the requirement for self-defense on one hand and the need for immunologic tolerance on the other. There is still much to understand about tolerogenic versus immunogenic forms of microchimerism, both of which have been reported [24].

 Taken together, these different observations a question may be raised whether the sequence of events in autoimmunity is at least partly due to alloimmunity rather than autoimmunity.

## 4. Pregnancy and Autoimmune Diseases

It is well recognized the highest prevalence of autoimmune diseases in childbearing-age women than in men. It is also well known that autoimmune diseases have a profound influence on pregnancy outcome [19, 25–27]. Hormonal and genetic factors are probably involved, but a clear explanation for this type of preponderance is currently lacking. However, the role of fetal microchimerism as a contributing factor for the starting or maintenance of the autoimmune reaction in women is an attractive hypothesis [1].

 Fetal cells have been found not just in peripheral blood but also within a variety of damaged tissues, where the autoimmune reaction is taking place [11, 28–31]. The presence of activated immune fetal cells within the maternal tissues may trigger susceptible women to develop autoimmune disorders. As the placental-induced immune suppression is vanished, the fetal immune cells may indeed become activated and initiate the autoimmune reaction [19]. The cause of this relationship has been based on the degree of HLA discrepancy between host and alien cells which may determine the status of any potential graft versus host reaction.

 On the other hand, it should be emphasized that a positive effect of pregnancy on autoimmune disease is generally observed. As previously mentioned, the placenta induces immune suppression and so lessens autoimmune activity. Actually, the amelioration of autoimmune clinical manifestations along gestation is a usual clinical observation. Experimental data suggest that, despite the relative enhancement of Th2 reaction, both arms of the immune response (Th1 and Th2) are globally reduced during pregnancy. The observation is supported by the increase in T-regulatory cells observed in pregnancy and because autoantibodies greatly decrease during pregnancy [32].

Assuming that part of microchimeric fetal cells could be progenitor cells of the fetal immune system, the pathogenic effect of fetal microchimeric cells in autoimmune diseases can be perpetuated. These cells could survive in bone marrow or move to maternal different organs, where they could proliferate, differentiate, and activate. The activation of fetal immature T cells, monocytes, macrophages, and natural killer cells and the production of inflammatory cytokines and chemokines are believed to initiate then autoimmune diseases [33, 34]. Alternatively, these cells could be recognized as partially alloimmune and in consequence give rise to the autoimmune reaction [10].

 Finally, this potential association is also supported by the observation that, albeit with few exceptions, the resetting to normal immune status in the postpartum period is usually coincidental with a clinical exacerbation of many autoimmune diseases.

## 5. Microchimerism and Autoimmune Thyroid Disease

 Several evidences have shown that female subjects with autoimmune thyroid diseases frequently have microchimeric fetal cells residing within their thyroid glands [3, 11, 12]. This has been described both in animals [27, 35] and in humans [11, 12, 14, 36, 37]. Whether the presence of fetal cells increases maternal thyroiditis or a previous episode of thyroiditis increases the recruitment of these fetal cells remains unknown. A number of studies revealed this association demonstrating that the prevalence of male cells is higher in women with autoimmune thyroid diseases who previously had given birth to a son than in women without autoimmune thyroid diseases who previously had given birth to a son [10, 14]. These results generate the attractive hypothesis of a causal relationship between microchimerism and autoimmune thyroid diseases.

 Davies' laboratory has extensively investigated the influence of pregnancy in autoimmune thyroid diseases, including the relationship between fetal microchimerism and autoimmune thyroid diseases [27, 35]. The group initially found that experimental autoimmune thyroiditis in mice enhanced the accumulation of intrathyroidal fetal cells during pregnancy [35]. The murine model of experimental autoimmune thyroiditis was established in female mice using murine thyroglobulin (Tg) as antigen. Tg-treated mice developed a florid lymphocytic infiltration by 4–6 weeks after immunization [27]. In addition, the results showed that there were no significant differences in thyroid function between nonimmunized and Tg-immunized pregnant mice.

 Davies' group, going forward in the study of fetal microchimerism as a cause involved in the development of autoimmune thyroid disease, used their experimental autoimmune thyroiditis model using sex-determining region Y (SRY) gene as the marker of presence of male microchimeric cells within maternal thyroids. The results demonstrated the presence of fetal cells in 46% of Tg-immunized pregnant mice, whereas few male fetal cells were detected in only 20% of controls or nonimmunized pregnant mice. Subsequent studies of cell characterization revealed the immune origin of the cells that accumulate within the thyroid of mice with experimental autoimmune thyroiditis during pregnancy and early postpartum [35]. These fetal cells were identified as regulatory and cytotoxic CD4+ (in a significant proportion), CD11c+, and weakly CD8+, but not from B220/CD45R+, CD11b+, or Sca-1+, which indicates that intra-thyroidal fetal cells included T-cell and dendritic cell lineage. This was accompanied by high titers of antibodies to Tg. Furthermore, mice postpartum followup revealed that intra-thyroidal fetal cells were most easily seen in experimental autoimmune thyroiditis animals during pregnancy, whereas the presence of these cells decreased in the postpartum period. So, a fetal cell-induced modulation during pregnancy and postpartum was highly feasible.

 As aforementioned, several clinical studies have found male cells in thyroid samples of women previously diagnosed of Hashimoto's thyroiditis [12–14, 37]. The presence of alien cells in autoimmune involved thyroids ranged between 38 and 83%. This wide spectrum of percentages probably reflects the discrepancies in study design. Interestingly, the percentage differences between the Hashimoto's thyroiditis and control groups in individual studies are constant. Although the presence of fetal microchimerism was not identified in normal thyroids or patients with nodular goiters by all authors [12, 13], nowadays it is considered that microchimeric cells are present in normal glands and around 20% of follicular adenomas [10]. A recent Italian report has revealed fetal microchimeric cells in normal maternal thyroid tissue [38]. The authors explain the discrepancy due to the different origin of normal samples, from the normal tissue contralateral, to a neoplastic lesion. This finding further supports the idea that, in the presence of a neoplastic process, microchimeric cells could migrate to the thyroid and participate in the repair process [38]. All in all, these results indicate a higher number of fetal microchimeric cells in autoimmune thyroid diseases than in normal thyroids or benign proliferative disorders [11, 14, 19, 37]. Therefore taken together, these findings strongly support a possible pathogenic role for fetal microchimeric cells in the development autoimmune thyroid disease. However, there is still an important missing link that is the presence of maternal microchimerism in male patients with Graves' disease, which has been poorly studied.

## 6. Fetal Microchimerism and Graves' Disease

### 6.1. Graves' Disease in Pregnancy

Pregnancy-related factors have a strong influence in Graves' disease [25]. Normally, the clinical course of Graves' disease improves as pregnancy progresses, paralleling the reduction in serum TSH receptor (TSH-R) autoantibodies level. The reflection of this clinical improvement could be not only the quantity of serum antibody concentration but also the quality (or the biological action) of these antibodies. Some authors have hypothesized that the equilibrium between TSH-R antibodies blocking and stimulating activities may shift in favor of blocking antibodies [39], though not all the experts share this opinion [40]. In any event, a fluctuant Graves' disease clinical evolution is a common finding during pregnancy with exacerbation during approximately the first three months and improvement in the last trimester [41].

 In the postpartum period as the pregnancy-associated immune-privileged state disappears, a relapse, exacerbation, or new onset of Graves' disease may occur. This outbreak normally happens between 4–12 months after delivery [42]. In fact, epidemiological studies show that around 60% of childbearing age women develop Graves' disease during the first year after delivery [29, 43], whereas the frequency of relapses varies from 30% to 70% of cases [44]. Likewise, an increased risk of developing Graves' disease after pregnancy may be greater in older patients (>35 years), and this risk lasts for several years after delivery [21, 45].

### 6.2. Experimental Evidence

It was also Davies' group who in 2002 first demonstrated that intrathyroidal fetal microchimerism was common and profound in female patients with Graves' disease [11]. Renné et al. confirmed these findings two years later [14]. Since then, more information has arisen in the scientific arena [10].

 Davies' group research was conducted in a sample of 27 thyroid glands from patients with a past medical history of Graves' disease [11]. The investigators analyze the presence of male-specific SRY gene in maternal thyroid glands by ELISA-PCR technique for the detection of DNA. This was a two-step designed study. Initially, male cell assay was applied to screen for circulating peripheral blood fetal microchimerism in 20 females and lastly in stored thyroid tissue. The preliminary results showed that none of 16 never-pregnant females had male cells detected. However, in previous pregnant women, male cells were easily detected in 28.6% of blood samples. Furthermore, 47% of female Graves' blood specimens contained significant male cells. These results were similar to prior reports indicating that peripheral blood fetal microchimerism is a common finding in childbearing-age women.

 A subsequent analysis was focused on thyroid samples from females previously diagnosed of Graves' and in a group of thyroid adenomas as control. The storage of the thyroid samples had been diversed. Twenty glands had been prepared in paraffin blocks, whereas the remaining seven were frozen. Interestingly, SRY gene was found in only 20% of paraffin embedded tissues, while 86% of the frozen samples were positive for the Y chromosome gene. In the former group, the ratio of male to female cells ranges from 14 to 295 by 10^5^, with a median of 37/10^5^ male/female cells. SRY gene was searched in 10 thyroid adenoma specimens (6 in paraffin and 4 frozen). None of the 6 paraffin-embedded thyroid adenoma was positive for the SRY gene analysis, whereas 1 out of 4 frozen samples with thyroid nodules showed male cells. Authors speculate that the greater detection of the SRY gene in frozen female thyroid tissues was probably due to DNA fragmentation in the paraffin-derived samples. Unexpectedly, many of the patients with male cell-positive thyroids had no history of earlier male pregnancies at the time of surgery. However, as the authors stated in the discussion, this did not necessarily exclude the possibility of undetected first trimester pregnancies because it has been demonstrated that fetal microchimerism can be established in the first month of pregnancy [11, 46].

Renné et al. study compared three entities: Graves' disease, Hashimoto's thyroiditis, and nodular or diffuse follicular adenomas from women whose childbirth history was positive for sons [14]. These investigators screened by fluorescence *in situ* hybridization for X chromosome and Y chromosome specific staining from paraffin-embedded thyroid specimen taken at surgery. The results showed that 23 out of 49 thyroids (47%) were positive for Y-chromosome-specific staining. These authors found no Y-chromosomes-positive thyrocytes. The proportion of Y-chromosome-positive thyroid section was highest in Hashimoto's thyroiditis (15/25; 60%), lower in Graves' disease (6/15; 40%), and infrequent in follicular adenomas (2/9; 22%) [14]. The results indicate a higher degree of microchimerism in autoimmune thyroid disease than in benign proliferative disorder. The results were consistent with previous reports also showing that microchimeric cells are found in more subjects with autoimmune thyroid diseases than in any other thyroid diseases [11, 13].

A summary of these findings is presented in Table 1.

## 7. Controversial Studies about the Relationship between Microchimerism and AITD

 Those dissenting from the appraisal of the relation between autoimmune thyroid disease and microchimerism mainly base on data from epidemiological studies. An Australian study over a large community-based female population (1,045 participants) showed no association between parity and presence of thyroid autoantibodies or thyroid dysfunction. Hence, the authors suggested a lesser role of fetal microchimerism in autoimmune thyroid diseases [47]. A year later, a Danish group also investigated the association between the presence of circulating thyroid autoantibodies and previous pregnancy, parity, and the use of estrogens in an even larger population cohort of 3,712 women. These authors reinforce the Australian conclusion, as they also did not find any association between thyroid antibodies and pregnancy. No association was observed between hormonal replacement therapy and serum TPO antibodies levels either. In line with the Australian authors, the Danish investigators concluded that there was no association between previous pregnancy and serum thyroid Abs level, which argues, in their opinion, against the role of microchimerism as a trigger factor of thyroid autoimmunity [48]. These two studies seem to indicate that the risk of having TPO antibodies or Tg antibodies was similar in nulliparous women compared with women with one or more previous pregnancies. However, recently in 2011, an American study was published with a confronting conclusion [49]. The authors analyze the relationship between TPO antibodies and increasing parity in a population of 17,298 women, larger than the two previous samples. The authors analyze the relationship of serum TPO antibody levels and increasing parity. Despite that the incidence of abnormally elevated TPO antibody levels increased with advancing parity, this trend was not significant after adjustment for maternal characteristics. However at higher TPO antibody levels, a significant relationship with advancing parity persisted after adjustments. Therefore, it was concluded that advancing parity is associated with an increased risk for high serum concentrations of TPO antibodies, suggesting that fetal microchimerism may play a role in development of autoimmune thyroid disorders [49]. The two main results from this large American study could help to explain why Australian and Danish authors could not find any relationship between parity and presence of TPO antibody levels. Nevertheless, it remains to be demonstrated that the establishment of microchimeric cells openly influences the natural history of autoimmune thyroid diseases.

## 8. Conclusions

 Currently, there is a large body of evidence showing that maternal thyroid gland is a significant organ where a variety of fetal cells settle and persist for decades. These foreign immune cells may be activated after delivery once placental immune suppression is over. This remains an attractive hypothesis for the postpartum increase of prevalence of Graves' disease.

## Figures and Tables

**Table 1:** Summary of the main experimental and clinical findings of Graves' disease and microchimerism.

Thyroid tissue origin	Storage	*N*	Gene and technique	Presence of microchimeric cells (%)	Reference
Graves' disease	Paraffin-embedded	20	SRY gene by ELISA-PCR	20%	Ando et al. [11]
	Frozen	7	SRY gene by ELISA-PCR	86%	Ando et al. [11]
	Paraffin-embedded	15	X and Y chromosomes by FISH	40%	Renné et al. [14]

Thyroid adenoma	Paraffin-embedded	6	SRY gene by ELISA-PCR	0%	Ando et al. [11]
	Frozen	4	SRY gene by ELISA-PCR	25%	Ando et al. [11]
	Paraffin-embedded	9	X and Y chromosomes by FISH	22%	Renné et al. [14]

Hashimoto's thyroiditis	Paraffin-embedded	25	X and Y chromosomes by FISH	60%	Renné et al. [14]

SRY: sex-determining region Y; PCR: polymerase chain reaction; FISH: fluorescence in situ hybridization.
